# Mechanical and wetting properties of coated paper sheets with varying polydimethylsiloxane molecular masses in the coating formulation

**DOI:** 10.3906/kim-2104-57

**Published:** 2021-12-31

**Authors:** Çağla SÖZ

**Affiliations:** Department of Material Science and Technologies, Faculty of Science, Turkish-German University, İstanbul, Turkey

**Keywords:** Cellulose, hybrid materials, packaging, paper substrate, polydimethylsiloxane, surface wetting

## Abstract

Coated paper sheets were prepared by immobilizing a thin coating layer of cross-linked polydimethylsiloxane (PDMS) and inorganic particles onto Whatman filter paper Grade 1 (WFP) substrates. Several coatings that differed in terms of their PDMS molecular masses were sprayed onto WFP substrates to investigate the effect of this variation on the (i) wettability and (ii) mechanical properties of the samples. Different samples having clay or silica particles in the coating formulation were investigated separately. Nonwettable coated paper sheets with promising mechanical properties were achieved and further investigated in terms of (iii) thermal resistance.

## 1. Introduction

Studies on hybrid materials has attracted interest in both academic and industrial research for more than 30 years [1].Hybrid materials are composites composed of inorganic and organic building blocks blended and interacting at themolecular or nanoscale level. Hybrid materials possess not only the properties of the inorganic and organic parts in theirstructure but also the synergistic properties created by the presence of a large hybrid interface [1–3]. So, they can beregarded as multifunctional materials and found or prepared in form of thin or thick films, coatings, fibers, powders,foams, monoliths, and complex architectures [1,3]. In literature, hybrid materials are commonly classified according tothe nature of their organic-inorganic interfaces: Class I hybrid materials possess weak interactions between the interfaces,such as van der Waals forces, hydrogen bonds or weak electrostatic interactions. Class II hybrid materials, on the otherhand, show strong chemical interactions between the organic and inorganic components [1,3]. Although the differencebetween hybrid materials and (nano)composites is vague, hybrid materials can be thought as a special class of (nano)composites, in which the high interface between organic and inorganic constituents and the new emerging synergisticproperties are pronounced [3]. These materials have many application areas such as energy-storage, electrocatalysis,display units, photovoltaics, energy-conversion systems, proton-pump electrodes, sensors, chemiresistive detectors,selective membranes, and also food packaging [1,2].

An important branch of hybrid materials is paper-based hybrid materials. Studies on paper-based hybrid materials are frequently reported, in which their promising application areas as electrode materials, mechanically improved films or filtration membranes are mentioned [4–6]. These studies reveal that, apart from its conventional usage as information storage medium, tissue paper or packaging material, paper is a highly promising substrate for advanced materials. Paper is composed of cellulose, a very abundant, renewable, and natural polymer it possesses a porous structure composed of fibers, has surface functional groups, and is inexpensive and widely available. All of its advantages make paper a special substrate to work on [4,6].

In this study, coated paper sheets were composed of paper substrate and a coating layer possessing inorganic particles embedded in a PDMS matrix. Clearly, paper is the organic component whereas the particles are the inorganic components of the material. PDMS, on the other hand, is known to have both inorganic and organic properties: The -Si-O- backbone is regarded to have an inorganic nature whereas the methyl substituents attached to Si, are the organic groups [7]. So, the coated paper sheets of this study are composed of organic and inorganic moieties at molecular scale. Since it was revealed by SEM and EDS investigations of previous studies that the samples prepared through the reported method possess the polymeric part not only on the surface but also throughout the crosssection of the paper substrate resulting sheets composed of nonseparable PDMS polymer and cellulose fibers, the samples are though as a kind of hybrid paper sheets [8]. Generally speaking, the samples described in this study are representatives of paper-based materials.

Such finished papers can in principle be obtained by applying a coating formulation consisting of organic matrix and inorganic fillers to the paper substrates using methods such as dip coating [9–12], drop coating [13], electrospraying [14], sol-gel processes [10,11] or brush coating [11]. The methods are either single-step [9,10,14,15] or multistep [11–13,16] modification processes. Moreover, studies on preparation of paper-based packaging materials or shopping bags occupy a large place in the literature [17–20]. In the studies, it has been reported that colloidal silica particles obtained by Stöber method [11,13,16], pyrogenic fumed silica fillers produced in flame [14,15] or platelet shaped clay particles [17,18,21–24] were added to the coating layer. The studies report on making paper surfaces hydrophobic exclusively by means of polymer coatings [9]. However, composite coating layers possessing both polymeric matrix and filler particles are needed to achieve superhydrophobicity on paper surfaces [10–16].

In our previous study, a coating mixture of PDMS and inorganic particles was covalently attached onto WFP through an easy to implement, and reproducible procedure to prepare superhydrophobic paper sheets [24]. So, a highly practical alternative to the existing preparation techniques of paper-based materials was introduced which also enabled the preparation of chemically robust sheets with an immobilized coating layer. Recently, we reported that this procedure is applicable for preparation of packaging materials [25]. In this study, which can be regarded as a continuation of the previous works, the effect of PDMS molecular weight in the coating formulation on (i) the wetting behavior and (ii) the mechanical properties of the samples were investigated besides reporting (iii) the thermal resistance of the mechanically optimized coated paper sheets.

## 2. Materials and methods

### 2.1. Materials used in the study

Commercial Whatman filter paper (WFP) Grade 1 was purchased from General Electric (Turkey). Trimethylsiloxy-terminated polydimethylsiloxane (PDMS) (silicone oil) with molecular masses of 1250 g/mol (PDMS-1.3K), 5970 g/mol (PDMS-6K), 9430 g/mol (PDMS-9K), 49350 g/mol (PDMS-50K), and 91700 g/mol (PDMS-92K) were purchased from Gelest (Morrisville, PA, USA). Glass spheres (GS), montmorillonite (MMT) and kaolin (K) were purchased from Sigma Aldrich (Turkey). Aeroperl 300 Pharma colloidal silica (aero) was kindly supplied by Evonik (İstanbul, Turkey) via Marmara Ecza (İstanbul, Turkey). HDK N20 fumed silica was kindly supplied by Wacker (İstanbul, Turkey) via IMCD Group (İstanbul, Turkey). Reagent grade tetrahydrofuran (THF) was purchased from Merck (Germany) and used as received.

### 2.2. Preparation of the samples

Sample preparation procedure was explained in the previous studies [24,25]. Briefly, PDMS oil was dissolved in THF (0.7 wt%) by magnetic stirring so that a dilute solution was obtained. Inorganic particles were added into the PDMS solution, so that the ratio of the inorganics to PDMS was 3/1 in weight/weight. The mixture was stirred and sonicated and was applied through spray-coating onto WFP substrates. The samples were then left in the hood overnight for the evaporation of the THF solvent. Afterwards, heat treatment at 120 °C for 36 h was performed. When the samples were cooled down to 23 °C, Soxhlet extraction with THF was performed for 3 h to get rid of the unreacted PDMS.

### 2.3. Mechanical tests

Dry and wet tensile strength of samples was measured with a Zwick 4831 Z2.5 Stress-Strain Instrument according to TS EN ISO 1924-2 and TS 5163 ISO 3781 (Finch method) test standards. Test conditions were determined according to standard TS 636 EN 20187. Test strips with widths of 12.5 mm and lengths of and 50 mm were prepared for dry tensile strength measurements whereas the dimensions of the test strips kept at 12.5 mm and 90 mm for wet tensile strength determination. At least 5 measurements were performed for both the machine direction (MD) and the cross direction (CD) of the samples. The tensile strength values were divided by the average grammage of each sample to achieve the tensile index values, as shown in Eq. (1).


(1) $$\text{Tensile index }[\text{Nm}/\text{g}]=\text{tensile strength }[\text{N}/\text{m}]/\text{grammage }[\text{g}/{\text{m}}^{2}]$$

The relative wet strength was calculated by using the dry and wet tensile strength values according to Eq. (2):


(2) $$\text{Relative wet strength in }\%=\frac{\text{wet tensile strength}}{\text{dry tensile strength}}\times 100$$

The burst strength values of the samples were determined according to test standard TS 3124 ISO 2758 and recorded in kPa. The average of at least 5 measurements were taken per sample.

Folding endurance tests were performed with a Schopper type folding endurance tester according to the Tappi Standard T 423 cm-98. Ten measurements were performed for each sample. The folding endurance values were recorded as the logarithm of the number of double folds.

### 2.4. Gravimetric analysis

In order to determine the grammage of the coated papers and WFP, gravimetric analyses were performed. Ten samples with sizes of 2 × 2 cm^2^ were prepared for each coated paper sheet and WFP, respectively. Samples were put into the vacuum oven at room temperature for 24 h. After 24 h, samples were weighed and average of 10 weights were taken for each sample. The measured weight divided by the sample area (0.0004 m^2^) was recorded as the grammage of each sample. Moreover, the difference in average grammage values of coated paper sheet and WFP were recorded as coating thickness in g/m^2^.

### 2.5. Thermal aging studies

Thermal aging tests of the coated paper sheets were carried out similar to the tests reported in literature [26]. The samples with 2 × 2 cm^2^ dimensions were kept at 100 °C and 200 °C for 30 min, 1 h and 3 h. Then, visual detection of temperature and time dependent changes of the color and size of the coated paper sheets were performed. CA and CAH values of the sheets were also recorded.

### 2.6. Static and dynamic contact angle measurements

Static and dynamic contact angle (CA) measurements were performed with a Dataphysics OCA 15 goniometer equipped with SCA 20_U software. Prior to measurements, samples were conditioned for 24 h at 23 ± 2 °C and 50 ± 5% relative humidity. Sessile drop method was used for the static contact angle (CA) measurements: 10 μL triple distilled deionized water was dropped onto the sample surface, and the CA was measured. Ten measurements were performed for each sample surface and the average static CA value was calculated accordingly. Embedding needle method was selected for the measurement of dynamic CA values: 1 μL distilled and deionized water droplet was injected at a speed of 0.2 μL/min from the needle tip so that it touched the surface of the sample. Afterwards, volume of the droplet advancing water contact angle (θ_adv_). Right afterwards, the volume of the water drop was reduced down to its initial volume, 5 μL, with a speed of 0.2 μL/min without retracting the needle. The lowest CA value during that process was recorded as the receding water contact angle (was increased to 5 μL at a speed of 0.2 μL/min. The volume of the 5 μL drop was further increased to 25 μL with a speed of 0.2 μL/min without retracting the needle. The highest CA value observed was recorded during that volume increase as the θ_rec_). The contact angle hysteresis (CAH) value was determined as θ_adv_ - θ_rec_.

### 2.7. Determination of the optical properties

The CIE L*a*b* values of the paper substrates were measured according to the ISO2470-2 testing standard with the Konica Minolta Spectrophotometer CM-700d.

## 3. Results and discussion

### 3.1. Preparation of the coated paper sheets

In this study, coated paper sheets were prepared by spray-coating WFP surfaces with a mixture of PDMS and inorganic particles. The weight ratios of the ingredients in the coating formulation were kept constant whereas the molecular mass of the PDMS was varied in each coating, as summarized in Table 1. Sample with the code WFP/PDMS-1.3K/GS/aero/N20 is, for example, the sample prepared through spray-coating of Whatman Grade 1 filter paper with a mixture of trimethylsiloxy-terminated polydimethylsiloxane with a molecular mass of 1250 g/mol, glass spheres, colloidal silica, and fumed silica in THF, which was then processed with heat treatment and Soxhlet extraction steps.

The study aims to understand the effect of PDMS molecular mass in the coating formulation on (i) the wetting behavior and (ii) the mechanical properties of the resulting hybrid paper sheets and furthermore to evaluate (iii) the thermal resistance of the mechanically optimised samples. WFP was the reference material of the study. So, its properties were also tabulated. WFP was selected as the substrate for the coated sheets because of being composed of pure cellulose fibers and thus having high number of reactive hydroxyl groups on its surface. The knowledge gained in this study will be applied in the future for coating of different paper types selected according to the possible application area.

**Figure 1:**
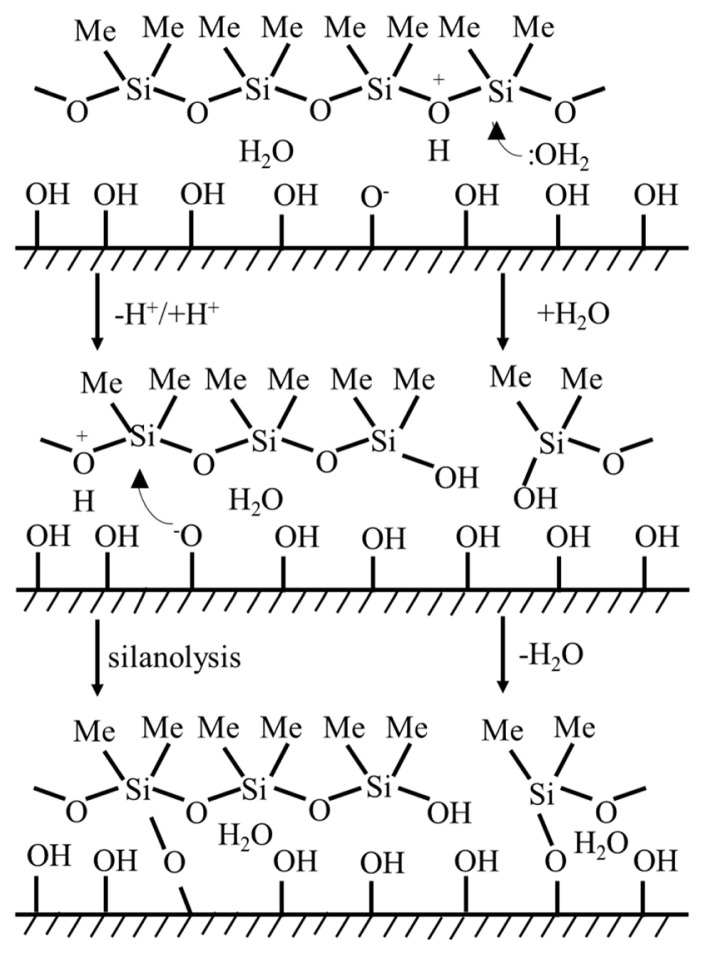
The possible mechanisms of Si-O-C bond formation on the paper substrate surface: an acid catalyzed silanolysis (left) or hydrolysis followed by condensation (left). Adapted from Ref. 27. Copyright 2011 American Chemical Society.

The thickness of the coating layer applied was also determined according to simple gravimetric studies. The difference in average grammage values of coated paper sheets and the WFP substrate was recorded as the coating thickness in g/m^2^, which was pretty similar irrespective of the formulation of coating layer and found to be 5.8 ± 1.7 g/m^2^. As mentioned in our previous study, it was inspired by the studies of Botter et al., Soares et al., and Krumpfer and McCarthy for immobilizing the coating layer on the paper substrate surface [27–29]. Briefly, trimethylsilyl-terminated polydimethylsiloxane (PDMS) chains, also known as silicone oil and considered as nonreactive, were used for covalent attachment to both the paper substrate and filler particles with hydrophilic and slightly acidic surface properties. The possible mechanisms proposed to explain the covalent bonding, are as follows: (i) the water-assisted hydrolysis of PDMS followed by condensation with hydroxyl groups on the substrate surface (right) and (ii) the direct or acid-catalyzed silanolysis of PDMS by the hydroxyl groups on the substrate surface (left), which are schematically represented in Figure 1 [27]. The schematic, adapted from the work of Krumpfer and McCarthy, is also suitable to reveal the possible mechanisms between hydrophilic and slightly acidic paper substrate and the PDMS domains: The hydroxyl groups on the paper substrate surface and siloxane units of PDMS take part in the reaction, resulting in Si-O-C bond formation. The heat treatment process, required for the covalent attachment of the coating onto the paper substrate, was optimized in previous studies. Briefly, without or by insufficient heat treatment, the coating layer on the paper substrate can be washed away through Soxhlet extraction in THF or hexane, whereas heat treatment at 120 °C for 36 h resulted in immobilization of the coating which resists the Soxhlet extraction procedure and also preserves the superhydrophobic nature.

In the above listed studies, Raman and FT-IR spectroscopies were used to reveal the covalent attachment between PDMS chains and the filter paper substrate. Briefly, Raman studies were performed to model compounds of microcrystalline cellulose (MCC) and PDMS, which were heat treated at 120 °C for 36 h. Peaks at 1161 cm^−1^ and 806 cm^−1^ were detected in Raman spectra of model compounds, which were absent in spectra of MCC or PDMS and were attributed to the Si-O-C asymmetric and asymmetric stretching peaks, respectively [24]. Additional studies based on FT-IR spectroscopy revealed the disappearance of the peaks centered at 1053 cm^−1^ and 1031 cm^−1^ for coated paper sheets, namely WFP/PDMS-6K/GS/aero/N20, which correspond to C-OH stretching of the secondary alcohol and C-OH stretching of the primary alcohol in the cellulose backbone of the WFP substrate [25].

### 3.2. Wetting properties

Wetting properties of WFP and coated paper sheets were listed in Table 2. As already known in literature, hydrophilic surfaces have CA values lower than 90°. Hydrophobic surfaces, on the other hand, have CA values greater than 90°. Superhydrophobic surfaces, surfaces that are nonwettable, have CA values greater than 150° and contact angle hysteresis (CAH) or tilt angle values below 10° [24,30]. CAH values were determined for those samples which have CA values above 150°, so that superhydrophobicity could be proved.

As shown in Table 2, all the coated samples had CA values above 150° and CAH values below 10° revealing that they are all superhydrophobic. So, WFP, which is an inherently hydrophilic material and adsorbs water droplets within seconds could be transferred to nonwettable, irrespective of the PDMS molecular mass in the coating formulation. It is important to point out that the WFP/PDMS samples without inorganics in the coating layer were also prepared and their CA values were measured. The PDMS content in the coating formulation was kept the same to be able to compare the outcomes: Briefly, PDMS was dissolved in THF (0.7 wt%) by magnetic stirring and subsequent ultrasonication steps. The dilute solution was then spray-coated onto the paper substrates. The CA values were not tabulated because they were regarded as 0° because of speed absorption of the water droplets by the paper substrate within seconds, irrespective of the PDMS molecular weight in the coating formulation. The reason is the nature of paper substrate: Paper is a porous nonwoven material composed of cellulose fibers. The voids of porous paper material reach diameters up to several tens of microns. When a dilute PDMS solution is applied onto the paper substrate surface, the PDMS layer does not fill these voids and water droplets could still be adsorbed by the paper substrate through the capillary effect [31,32].

Besides the static and dynamic contact angle measurements, which are performed to reveal the nonwettability material surfaces, Cobb tests are also necessary to get an idea about the interaction of paper-based materials with water, which reveal the absorbency and thus the extent of water barrier properties of these materials [33–35]. Cobb tests of WFP, WFP/PDMS-6K/GS/aero/N20, and WFP/PDMS-6K/MMT/K/N20 were determined in previous studies to prove the high water properties of the coated samples especially when compared to that of WFP [36]. Briefly, the mass of water absorbed by one square meter of the coated paper sample at a given time were calculated and found to be 220.2 ± 0.3 g/m2 for WFP, 47.8 ± 15.2 for WFP/PDMS-6K/GS/aero/N20 and 24.1 ± 7.1 for WFP/PDMS-6K/MMT/K/N20. The Cobb values of the coated paper samples, which were significantly lower than that of the WFP, revealed that the samples possessed high barrier properties against water besides being superhydrophobic since samples with water absorption values of 50 g/m^2^ or below are accepted as samples with high water barrier properties in paper industry [34,35].

### 3.3. Mechanical properties

#### 3.3.1. Tensile test results

**Figure 2:**
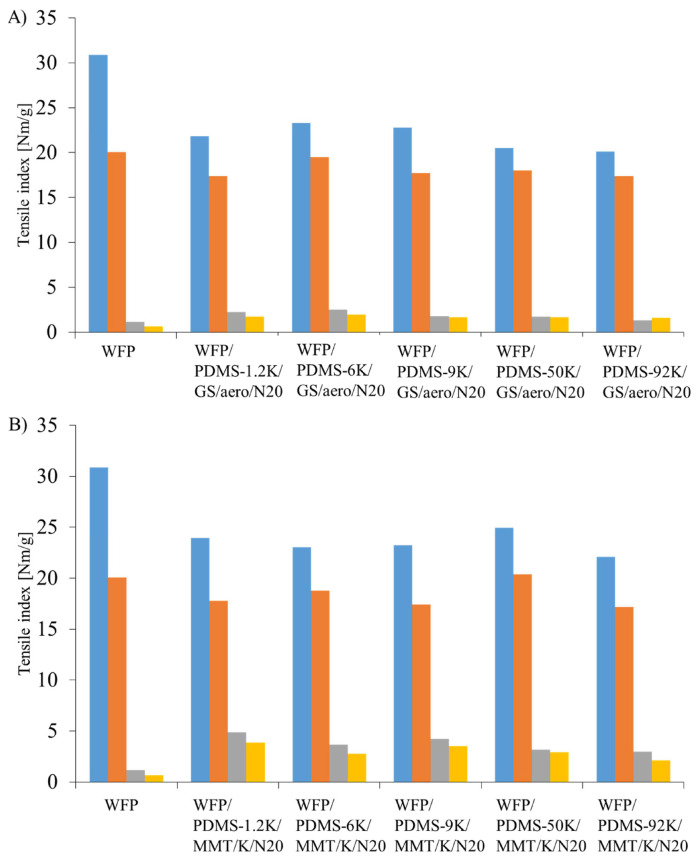
The dry and wet tensile indices of A) WFP/PDMS/GS/aero/N20 and B) WFP/PDMS/MMT/K/N20 in MD and CD.

For the determination of mechanical properties of paper and paper-based materials, tensile index measurements are frequently performed [12,37]. In this study, the effect of the coating layer on the tensile properties of the WFP substrate was investigated. Moreover, the effect of PDMS molecular mass in the coating layer on tensile properties of the coated paper sheets was studied. The effect of wet and dry states of the coated paper sheets on the final mechanical properties were also investigated. As already known in the literature, measurements in both machine and cross directions (MD and CD) are necessary for samples with commercial paper substrates because the differences of fiber alignment in both directions can have an impact on mechanical strength of the samples [12,37]. The dry and wet tensile indices and also the relative wet strength values of the samples in both directions are listed in Table 3. Tensile indices of the coated paper sheets were also revealed in Figure 2. The data were obtained according to Eq. (1), in which the tensile strength values of the samples were divided by the corresponding grammage value.

As revealed in Table 3, among samples having the composition of WFP/PDMS/GS/aero/N20, coating application slightly decreased the dry tensile index values of WFP in both directions. The sample having PDMS-6K in the coating formulation resulted in highest dry tensile index values in both directions among these coated paper sheets. The wet tensile indices of the coated paper sheets, however, are much higher than those of WFP, as expected. Similarly, the coated paper sheet having PDMS-6K in the coating formulation resulted in highest relative wet strength values in both directions. So, it was decided that the WFP/PDMS-6K/GS/aero/N20 sheet is the optimized sample among others having only colloidal and fumed silica particles in the coating formulations in terms of mechanical properties.

When the dry tensile strength values of the samples with the composition WFP/PDMS/MMT/K/N20 are considered, again a slight decrease was detected with respect to the dry tensile strength values of WFP. However, these samples have all a significant increases in wet tensile index values, which are much higher than those of WFP/PDMS/GS/aero/N20 samples. So, the coating layer with platelet-shaped particles in the formulation protects the paper substrate fibers from wetting significantly, so that higher wet tensile index values were detected. Accordingly, the highest relative wet strengths among all samples were observed for the WFP/PDMS/MMT/K/N20 samples. An effect of PDMS molecular weight on the mechanical properties could not be detected.

In this study it was also investigated whether the critical entanglement molecular weight (M_c_) of PDMS, which was reported to be approximately 24,500 g/mol, had any impact on tensile properties of the coated samples [38,39]. The results listed in Table 3 revealed that there is no significant contribution from PDMS molecular mass on the tensile properties of the coated paper sheets. The reason is thought to be the decrease in molecular weight of PDMS chains during curing steps on the paper substrate. In light of the previous studies, it is known that unbound PDMS or other moieties are not present on the sample surfaces: Coated paper sheets were subjected to Soxhlet extraction for 3 to 10 h to get rid of the unbound coating ingredients, as explained in previous studies [24,25]. Briefly, SEM studies were performed to investigate the coated paper sheet surfaces. After Soxhlet extraction procedure, the distinct coating topography with micro- and nanometer sized surface structures fully covering cellulose fibers of the paper substrate were detected on the sample surfaces. On the other hand, when the samples were prepared without heat treatment and subjected to Soxhlet extraction, the coating layer was washed away so that bare and fibrous substrate surface was revealed by SEM. So, coated paper sheets that were the subject of this study are thought to have covalently attached coating layers.

The relative wet strength values of the samples were calculated by using Eq. (2). The relative wet strength value of WFP is 3.7 and 3.4% in MD and CD, respectively. The coated paper sheets, on the other hand, have much higher relative wet strength values than those of the WFP. Among samples with the formulation WFP/PDMS/GS/aero/N20, the highest increase was detected for the coated paper sheet with PDMS-6K with a significant increase in relative wet strength values 10.5% (MD) and 9.8% (CD). The superhydrophobicity implemented by the coating had a positive impact on the wet tensile properties of the paper, as expected.

Increase in wet strength values is much more pronounced for the samples having the formulation WFP/PDMS/MMT/K/N20. PDMS molecular mass does not seem to have an effect on the relative wet strength values. The studies on mechanical properties of the coated paper sheets in general revealed that the coating layer application has a positive impact on the mechanical performance of the samples in wet state.

#### 3.3.2. Preliminary studies on burst strength and folding endurance

Preliminary studies on burst strength and folding endurance were performed by using WFP substrate as a control sample and the WFP/PDMS-6K/GS/aero/N20 paper sheet. The burst strength value for WFP was found to be 108.02 ± 0.79 kPa, whereas the burst strength for SH-WFP was measured as 95.96 ± 4.42 kPa, which revealed that the coating application did not have a positive effect on the burst strength of the control substrate. The folding endurance values, which are defined as the logarithm of the number of double folds, were measured to be 0.77 ± 0.13 and 0.97 ± 0.11 for WFP, and WFP/PDMS-6K/GS/aero/N20, respectively, which reveal a promising increase of 25.9% in terms of mechanical strength. Although the folding endurance measurement, a test used to specify the refoldability and quality of paper, is primarily preferred for testing books or banknotes through exposure to cyclic folding [40–42], there are also studies on folding endurances about packaging paper alternatives to reveal the durability of these materials when folded successively. It is known that higher folding endurance values are correlated to higher flexibility and higher mechanical properties [43–45]. At this stage, it is also important to point out that the ongoing studies on enhancing the mechanical properties of control samples and coated paper sheets continues.

### 3.4. Thermal ageing studies

**Figure 3:**
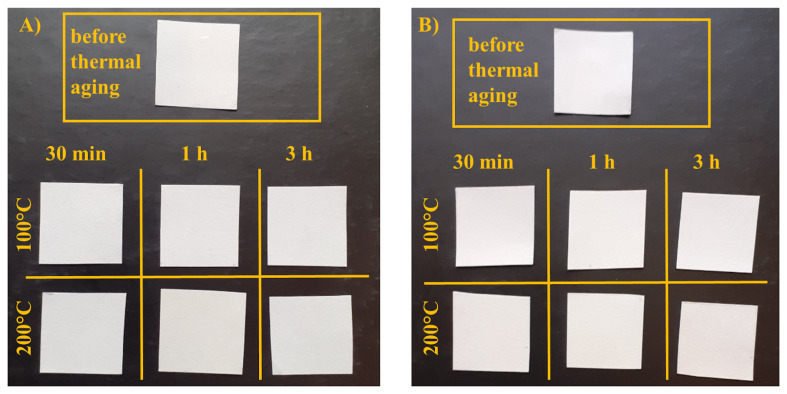
Appearances (changes in color and size) of the coated paper sheets A) WFP/PDMS-6K/GS/aero/N20 and B) WFP/PDMS-6K/MMT/K/N20 depending on temperature and time.

Thermal aging tests of paper-based composite and/or hybrid materials are frequently encountered in the literature to reveal the thermal durability of those [26,47,48]. The main purpose of thermal aging tests is to observe, whether there is a change in the properties of samples exposed to high temperatures for different periods of time, thus determining the working condition limits of samples [26]. In this study, thermal aging tests of WFP/PDMS-6K/GS/aero/N20 and WFP/PDMS-6K/MMT/K/N20 were conducted. The appearance of the samples after thermal aging is revealed in Figure 3 whereas the CA and CAH values are listed in Table 4.

As shown in Figure 3, coated paper sheets did not undergo any visible changes (shape, color and dimension) when stored up to 3 h at 200 °C. Sheets did also not lose their superhydrophobic property although a slight decrease in CA and CAH values were determined with increasing temperature and duration of the heat treatment. The results reveal that the coated paper sheets are thermally resistant even at 200 °C. These findings made us to conclude that the reported coating method, which is thought to be suitable for preparation of new packaging alternatives, is also suitable for the preparation of packages for hot goods such as bakery products.

### 3.5. Studies on the optical properties

CIE L*a*b* values of WFP, WFP/PDMS-6K/GS/aero/N20, and WFP/PDMS-6K/MMT/K/N20 are given in Table 5. Color differences (ΔE*) of the coated samples were also calculated [49,50]. WFP was selected as the control paper substrate whereas WFP/PDMS-6K/GS/aero/N20 and WFP/PDMS-6K/MMT/K/N20 are two of the promising samples of the study. CIE L* is known as the lightness value. It stands for black at 0 and white at 100. The a* values are present on an axis of green to red, on which negative values are towards green and positive ones are towards red color. The b* values, on the other hand, are on an axis of blue to yellow, on which with negative values are towards blue and positive values are towards yellow [42]. It was revealed in Table 5 that the lightness of the paper substrate slightly decreased upon coating application. As a result of the coating application, the sample color shifts slightly to green and slight yellowing was also detected. Both coated samples revealed color differences greater than 1, when compared to WFP. ΔE* value of WFP/PDMS-6K/MMT/K/N20 was less than that of WFP/PDMS-6K/GS/aero/N20.

### 3.6. Possible recycling routes for coated paper sheets

It is also important to point out the effect of covalent attachment of the coating layer to the paper substrate on the recycling conditions. For the potential packaging products prepared through the method proposed in this study, the best management of waste paper would be either burning for energy recovery. The samples prepared by the proposed procedure have the advantage of not containing additional metal catalysts like Pt, which are commonly used in the PDMS-based coatings like release liners, the major recycling procedure of which is incineration [51–53]. So, the packaging products will cause less environmental pollution in waste treatment step. Another option could be recycling of the samples in form of nonpaper products such as chipboard or cellulose insulation. Since the coating applied is chemically bound to the surface and also has the potential to complicate the deinking procedure, production of nonpaper products would be the most promising waste management method.

## 4. Conclusion

In this study, nonwettable, coated paper sheets with varying inorganic particle types and PDMS molecular masses were prepared. No significant changes were detected in wettability of the samples having PDMS with different molecular masses in the coating formulation. To preserve the comparability of the results, the PDMS and filler amounts in the formulation were kept constant, whereas the filler type and PDMS molecular weight were changed. However, future studies on the effect of the PDMS content in the formulation are planned. Similarly, PDMS molecular mass did not have a significant effect on the mechanical properties of the samples, especially those having the formulation WFP/PDMS/MMT/K/N20. In comparison with WFP, the coated paper sheets showed significant enhancements in wet tensile index in MD and CD, which was even more pronounced in sheets having platelet shaped clay particles in the coating layer. The thermal resistance of the WFP/PDMS-6K/GS/aero/N20 and WFP/PDMS-6K/MMT/K/N20 samples were also investigated, and samples were also found to be thermally resistant. The results revealed that the coating procedure proposed in this study might a promising alternative for the preparation of paper-based packaging materials, which, for example, might be suitable for the preservation food such as pizza or hot bakery products, when applied onto the suitable paper substrate.

## Figures and Tables

**Table 1:** List of the samples.

#	Sample code	mwt PDMS (gmol^−1^)	Filler type
1	WFP	-	-
2	WFP/PDMS-1.2K/GS/aero/N20	1250	glass spheres/colloidal silica/fumed silica
3	WFP/PDMS-6K/GS/aero/N20	5970	
4	WFP/PDMS-9K/GS/aero/N21	9430	
5	WFP/PDMS-50K/GS/aero/N20	49350	
6	WFP/PDMS-92K/GS/aero/N20	91700	
7	WFP/PDMS-1.2K/MMT/K/N20	1250	montmorillonite/kaolin/fumed silica
8	WFP/PDMS-6K/MMT/K/N20	5970	
9	WFP/PDMS-9K/MMT/K/N20	9430	
10	WFP/PDMS-50K/MMT/K/N20	49350	
11	WFP/PDMS-92K/MMT/K/N20	91700	

**Table 2:** CA and CAH values of samples.

#	Sample code	CA (°)	CAH (°)
1	WFP	0	-
2	WFP/PDMS-1.2K/GS/aero/N20	159.0 ± 1.8	7.2 ± 1.3
3	WFP/PDMS-6K/GS/aero/N20	161.9 ± 1.5	5.4 ± 1.0
4	WFP/PDMS-9K/GS/aero/N21	160.2 ± 1.1	6.8 ± 1.9
5	WFP/PDMS-50K/GS/aero/N20	161.1 ± 2.0	6.2 ± 1.4
6	WFP/PDMS-92K/GS/aero/N20	160.4 ± 1.2	7.1 ± 1.1
7	WFP/PDMS-1.2K/MMT/K/N20	162.0 ± 1.1	5.1 ± 1.7
8	WFP/PDMS-6K/MMT/K/N20	163.4 ± 2.0	3.4 ± 0.7
9	WFP/PDMS-9K/MMT/K/N20	161.8 ± 0.7	6.1 ± 1.8
10	WFP/PDMS-50K/MMT/K/N20	161.5 ± 0.3	6.9 ± 1.1
11	WFP/PDMS-92K/MMT/K/N20	161.7 ± 0.6	7.4 ± 1.3

**Table 3:** Tensile test results.

#	Sample code	Dry tensile index (MD) (Nm/g)	Dry tensile index (CD) (Nm/g)	Wet tensile index (MD) (Nm/g)	Wet tensile index (CD) (Nm/g)	Relative wet strength (MD) (%)	Relative wet strength (CD) (%)
1	WFP	30.68 ± 1.41	20.06 ± 0.62	1.17 ± 0.10	0.66 ± 0.02	3.7	3.4
2	WFP/PDMS-1.2K/GS/aero/N20	21.82 ± 0.45	17.39 ± 0.78	2.25 ± 0.59	1.73 ± 0.37	10.1	9.9
3	WFP/PDMS-6K/GS/aero/N20	23.31 ± 0.65	19.51 ± 1.28	2.49 ± 0.35	1.97 ± 0.49	10.5	9.8
4	WFP/PDMS-9K/GS/aero/N21	22.76 ± 0.51	17.70 ± 1.16	1.80 ± 0.35	1.77 ± 0.06	8.1	9.3
5	WFP/PDMS-50K/GS/aero/N20	20.53 ± 1.94	17.99 ± 1.82	1.72 ± 0.18	1.69 ± 0.41	8.6	9.2
6	WFP/PDMS-92K/GS/aero/N20	20.11 ± 0.97	17.35 ± 1.30	1.32 ± 0.35	1.61 ± 0.24	6.7	9.4
7	WFP/PDMS-1.2K/MMT/K/N20	23.93 ± 2.11	17.76 ± 1.12	4.88 ± 0.32	3.85 ± 0.84	20.6	21.4
8	WFP/PDMS-6K/MMT/K/N20	23.01 ± 1.69	18.77 ± 1.20	3.68 ± 0.03	2.79 ± 0.15	14.2	13.2
9	WFP/PDMS-9K/MMT/K/N20	23.22 ± 1.75	17.42 ± 0.35	4.20 ± 1.04	3.50 ± 1.41	18.3	19.9
10	WFP/PDMS-50K/MMT/K/N20	24.94 ± 5.43	20.36 ± 4.38	3.15 ± 1.14	2.90 ± 1.12	12.8	14.1
11	WFP/PDMS-92K/MMT/K/N20	22.10 ± 1.47	17.14 ± 0.77	2.95 ± 0.71	2.11 ± 0.28	13.2	12.4

**Table 4:** Temperature and time dependent CA and CAH values of the coated paper sheets.

Sample code	Wetting properties	Before thermal aging	Thermal aging at 100 °C			Thermal aging at 200 °C		
			30 min	1 h	3 h	30 min	1 h	3 h
WFP/PDMS-6K/GS/aero/N20	CA (°)	161.9 ± 1.5	162.0 ± 1.5	161.4 ± 1.4	162.6 ±1.1	160.6 ±2.0	162.0 ±0.7	161.0 ± 1.1
	CAH (°)	5.4 ± 1.0	6.5 ± 1.2	7.5 ± 0.9	7.5 ± 1.0	7.3 ± 0.6	7.6 ± 1.1	8.9 ± 0.8
WFP/PDMS-6K/MMT/K/N20	CA (°)	163.4 ± 2.0	163.1 ± 1.5	162.1 ± 1.4	163.0 ± 1.0	160.4 ± 1.0	160.5 ± 1.9	160.9 ± 1.2
	CAH (°)	3.4 ± 0.7	3.4 ± 1.0	4.7 ± 0.4	4.6 ± 0.8	7.2 ± 0.9	7.4 ± 1.1	7.2 ± 1.3

**Table 5:** Optical properties of WFP and the coated paper sheets.

Sample	L^*^	a^*^	b^*^	ΔE^*^
WFP	95.61	0.03	−1.28	-
WFP/PDMS-6K/GS/aero/N20	94.19	−0.02	3.77	5.25
WFP/PDMS-6K/MMT/K/N20	94.78	−0.05	1.83	3.22
